# Vaccines as alternatives to antibiotics for food producing animals. Part 1: challenges and needs

**DOI:** 10.1186/s13567-018-0560-8

**Published:** 2018-07-31

**Authors:** Karin Hoelzer, Lisa Bielke, Damer P. Blake, Eric Cox, Simon M. Cutting, Bert Devriendt, Elisabeth Erlacher-Vindel, Evy Goossens, Kemal Karaca, Stephane Lemiere, Martin Metzner, Margot Raicek, Miquel Collell Suriñach, Nora M. Wong, Cyril Gay, Filip Van Immerseel

**Affiliations:** 1 The Pew Charitable Trusts, 901 E Street NW, Washington, DC, 20004 USA; 2 Ohio Agriculture and Research Development Center, Animal Sciences, Ohio State University, 202 Gerlaugh Hall, 1680 Madison Ave., Wooster, OH 44691 USA; 3 Pathobiology and Population Sciences, Royal Veterinary College, University of London, Hawkshead Lane, North Mymms, Hertfordshire AL9 7TA UK; 4 Department of Virology, Parasitology and Immunology, Faculty of Veterinary Medicine, Ghent University, Salsiburylaan 133, B-9820 Merelbeke, Belgium; 5 School of Biological Sciences, Royal Holloway University of London, Egham, Surrey TW20 0EX UK; 6 Science and New Technologies Department, World Organisation for Animal Health (OIE), 12 Rue de Prony, 75017 Paris, France; 7 Department of Pathology, Bacteriology and Avian Diseases, Faculty of Veterinary Medicine, Ghent University, Salsiburylaan 133, B-9820 Merelbeke, Belgium; 8 Elanco Animal Health, 2500 Innovation Way, Greenfield, IN USA; 9 Merial, 29 Avenue Tony Garnier, F-69007 Lyon, France; 10 RIPAC-LABOR GmbH, Am Mühlenberg 11, 14476 Potsdam, Germany; 11 MSD, 2 Giralda Farms, Madison, NJ USA; 12 Office of National Programs, Agricultural Research Service, USDA, Sunnyside Ave, 5601 Beltsville, MD USA

## Abstract

Vaccines and other alternative products can help minimize the need for antibiotics by preventing and controlling infectious diseases in animal populations, and are central to the future success of animal agriculture. To assess scientific advancements related to alternatives to antibiotics and provide actionable strategies to support their development, the United States Department of Agriculture, with support from the World Organisation for Animal Health, organized the second International Symposium on Alternatives to Antibiotics. It focused on six key areas: vaccines; microbial-derived products; non-nutritive phytochemicals; immune-related products; chemicals, enzymes, and innovative drugs; and regulatory pathways to enable the development and licensure of alternatives to antibiotics. This article, part of a two-part series, synthesizes and expands on the expert panel discussions regarding opportunities, challenges and needs for the development of vaccines that may reduce the need for use of antibiotics in animals; new approaches and potential solutions will be discussed in part 2 of this series. Vaccines are widely used to prevent infections in food animals. Various studies have demonstrated that their animal agricultural use can lead to significant reductions in antibiotic consumption, making them promising alternatives to antibiotics. To be widely used in food producing animals, vaccines have to be safe, effective, easy to use, and cost-effective. Many current vaccines fall short in one or more of these respects. Scientific advancements may allow many of these limitations to be overcome, but progress is funding-dependent. Research will have to be prioritized to ensure scarce public resources are dedicated to areas of potentially greatest impact first, and private investments into vaccine development constantly compete with other investment opportunities. Although vaccines have the potential to improve animal health, safeguard agricultural productivity, and reduce antibiotic consumption and resulting resistance risks, targeted research and development investments and concerted efforts by all affected are needed to realize that potential.

**Electronic supplementary material:**

The online version of this article (10.1186/s13567-018-0560-8) contains supplementary material, which is available to authorized users.

## Introduction

Antibiotic resistance is a global threat to public health [1–4]. Every time an antibiotic is used in any setting, there is a risk of selecting for resistant bacterial strains [2, 4–6]. Therefore, prudent or judicious antibiotic use is important [7]. In animal agricultural production, that means using antibiotics only when absolutely necessary to protect the health of the animal and/or humans, relying on non-antibiotic alternatives to manage animal health where possible, and making optimal treatment choices with regard to antibiotic drug selection and treatment protocol when antibiotics are needed. Alternatives to antibiotics can help minimize the need for antibiotics by helping to prevent and control infectious diseases in animal populations. As such, safe and effective alternatives are crucially important to the future success of animal health and production. To assess scientific advancements in the research and development of alternatives to antibiotics, highlight promising research results and novel technologies, assess challenges associated with their commercialization and use, and provide actionable strategies to support their development, the United States Department of Agriculture (USDA), with support from the World Organisation for Animal Health (OIE), organized the second International Symposium on Alternatives to Antibiotics [8]. The symposium focused on six key areas: vaccines; microbial-derived products; non-nutritive phytochemicals; immune-related products; chemicals, enzymes, and innovative drugs; and regulatory pathways to enable the licensure and development of alternatives to antibiotics [9]. This two-part manuscript synthesizes and expands on the scientific presentations and expert panel discussions from the symposium regarding the use of vaccines as alternatives to antibiotics that can reduce the need for antibiotic use in animals. Part 1 synthesizes and expands on the expert panel discussions regarding the opportunities, challenges and needs related to vaccines that may reduce the requirement for use of antibiotics in animals, while part two focuses on highlighting new approaches and potential solutions. Other important factors relevant to the effective use of vaccines as alternatives to antibiotics, such as educational needs for producers and veterinarians, the combination of vaccination strategies with best management and husbandry practices, or behavioral aspects related to the adoption of vaccination practices are outside the scope of this manuscript and therefore not discussed here.

## Vaccines as alternatives to antibiotics

Vaccines are promising alternatives to antibiotics.^1^ In a recent multi-country expert-ranking of alternatives to the use of antimicrobial agents in pig production, vaccines were ranked highest for perceived feasibility and among the top five alternative approaches with regard to perceived effectiveness [10]. A quasi-experimental study of farrow-to-finish pig farms in Belgium has demonstrated the cost-effectiveness of enhanced biosecurity and vaccinations to reduce antibiotic consumption [11]. Similarly, the implementation of herd-specific action plans that included improvements in vaccination on pig operations in Belgium led to reduced antimicrobial consumption and improvements in production parameters such as mortality rates and daily weight gains [12].

A variety of studies have demonstrated that the use of various bacterial as well as viral vaccines in animal populations can result in a significant reduction in antibiotic consumption [13]. For example, the introduction and widespread routine use of a vaccine against *Aeromonas salmonicida* led to a significant decrease in antibiotic use in the farmed salmon industry [14, 15]. Similarly, research has demonstrated that vaccination against *Lawsonia intracellularis*, the causative agent of ileitis, in Danish pig herds can reduce oxytetracycline consumption for this condition by almost 80%; vaccination also led to significantly fewer pigs being treated with oxytetracycline, and improved productivity parameters such as average daily gains and carcass weights [16]. Improvements in mortality rates, feed conversion ratio, pig uniformity, the occurrence of clinical diarrhea, and the need for antibiotic treatment after *L. intracellularis* vaccination have also been reported, albeit the effects were in some cases relatively modest and statistical significance was not assessed in all of the studies [17–19]. Notably, in one study on 64 farms in 9 European countries, the majority of pig operations experienced cost reductions for antibiotic treatments after *L. intracellularis* vaccination, even though not all farms were able to reduce their antibiotic use [18].

In a study in Austrian pig herds, vaccination against porcine circovirus type 2 (PCV-2), a viral infection which leads to generalized immune suppression and therefore predisposes animals to secondary bacterial infections, led to a statistically significant decrease in antimicrobial consumption at the farm level, even though the impact varied significantly among farm types; while the impact on finishing farms was statistically significant the decline was negligible on farrow-to-finish farms [20]. The introduction of PCV-2 vaccination on a Dutch 460 sow farm resulted in improvements in average daily gain, mortality rates and decreased antibiotic use (measured as defined daily doses), assessed based on data spanning 8 months before vaccination, a 4 month transition period, and 12 months of routine vaccination [21]. Similarly, introduction of PCV-2 vaccination in a Canadian pig production system led to statistically significant improvements in attrition, average daily gains and mortality rates, leading to a reduction in antibiotic use and an estimated return on investment of 6.60 Canadian dollars for each dollar invested in vaccines, even though observations were restricted to a single operation and six production batches before and six after introduction of the vaccine [22]. In a Danish wean-to-finish pig herd, vaccination against both PCV-2 and *L. intracellularis* led to a considerable reduction in antibiotic consumption, improvements in average daily gains and mortality, and a 2.5–1 return on investment ratio [23]. In another study of Danish swine herds, the use of a vaccine against *Actinobacillus pleuropneumonia* resulted in a significant decrease in antibiotic consumption compared to non-vaccinated herds [24]. Similarly, vaccination against porcine reproductive and respiratory syndrome (PRRS) virus on a Belgian pig farm reduced antibiotic consumption by more than 50%, leading to a reduction in antibiotic cost by almost 50% [25].

Despite a dearth of quantitative studies, experts also generally agree that the use of vaccines has reduced the need for antimicrobial use in commercial poultry production [13]. In fact, a multi-center field trial of an avian colibacillosis vaccine in broiler chicken found significant differences in antibiotic consumption between vaccinated and control flocks, with consumption estimates averaging 0.5 treatment days for vaccinated and 2 days for unvaccinated flocks [26]. Other experimental studies have produced similar results [27]. Vaccination of broiler chicken may also confer additional benefits. Experimental evidence suggests that drug-sensitive parasite strains contained in coccidial vaccines and shed by vaccinated birds may aid in the restoration of sensitive parasite populations in the broiler house [28].

However, vaccination has not in all cases been associated with a decrease in antibiotic consumption. For instance, in one recent Danish study, pig herds that purchased vaccines against *Mycoplasma hyopneumoniae* and PCV2 had a significantly higher number of antimicrobial prescriptions compared with herds not purchasing these vaccines [29]. Similarly, a study of farrow-to-finish pig herds in Belgium, France, Germany and Sweden detected that antimicrobial consumption correlated inversely with the number of pathogens targeted with vaccines [30]. However, another study, a blinded field trial with two *M. hyopneumoniae* vaccines in Danish pig operations, failed to detect a statistically significant relationship between vaccination and antibiotic consumption or other relevant parameters, such as mortality or growth rates, although the prevalence of lung lesions was significantly reduced by one of the vaccines [31].

The reasons for the variable relationship between vaccination and antibiotic use in these studies have not been fully determined, but reinforce the complexity of research on the impact of vaccination on on-farm antibiotic consumption. One important factor may be potential systematic differences between vaccinated and control herds or flocks. For instance, a higher incidence of certain health problems may be a factor influencing operations’ vaccination decisions and therefore serve as a source of systematic bias [13]. This may, at least in part, explain the higher antibiotic consumption in some vaccinated compared to control operations, in particular if the vaccine is not able to completely control the spread of the disease in the population.

## Properties of current vaccines

Conventional veterinary vaccines include attenuated live vaccines and inactivated vaccines [32]. Live attenuated vaccines provide protection through a limited infection of a live organism which elicits an immune response, and may provide mucosal immunity [33–35]. The adaptive immune response elicited by live vaccines is composed of both humoral and cell-mediated responses, similar to that of a natural infection; this is in contrast to inactivated vaccines, which primarily stimulate a humoral response [34–36]. Inactivated or killed vaccines can be efficacious for providing protection against systemic infections and disease, but the protection provided by these vaccines has limited ability to prevent colonization on mucosal surfaces (e.g., in the intestine, urogenital tract, and respiratory tract) which are the most common portals of entry for pathogens [37, 38]. Additionally, these types of vaccine often depend on adjuvants and typically require injection of individual animals, which is not always practical. For instance, in the poultry industry in most regions of the world such approaches are not feasible, mostly due to large flock sizes and difficulties related to handling large numbers of birds.

For diseases caused by pathogens with multiple serotypes and serogroups, such as influenza or *Salmonella*, effective vaccination can be particularly challenging. For example, after vaccination, protection against homologous strains of *Salmonella* is high [39, 40], but often less protection is afforded against challenge by a heterologous serotype [35, 41]. Cross-serotype protection, in particular for minor serovars for which live attenuated vaccines are not available, has become one of the primary research focuses for *Salmonella* vaccines. Innovative new vaccine strategies are aimed at overcoming some of these challenges associated with conventional vaccines; they include marker vaccines, which permit distinction between naturally infected and vaccinated animals, as well as vectored, subunit and genetically engineered vaccines, and DNA vaccines [32].

Vaccines can be used to prevent or control infections in animal populations, or to minimize clinical signs and thus production losses after infection [32]. In rare cases, vaccines may also contribute to the eradication of a pathogen—as demonstrated for instance by the global eradication of rinderpest virus [42]. Conceptually, vaccines can reduce the threat of antimicrobial resistance development by preventing infections and thereby reducing the need to use antibiotics to treat primary bacterial infections or secondary bacterial infections following viral or parasitic infections. Moreover, vaccines may allow for the use of narrower-spectrum antibiotics by helping to rule out certain pathogens as the cause of a disease, and reduce disease pressures in populations by increasing herd immunity [43]. Potential vaccine effects on bacterial population densities and resulting resistance gene exchange rates have also been proposed [43].

## Limitations of current vaccines as alternatives to antibiotics

The ideal veterinary vaccine is safe, efficacious, and provides robust and durable protection against a broad spectrum of pathogens. At the same time, it must be easily administered, often on a large scale, and be cost-effective. However, many currently available veterinary vaccines have limitations that reduce their usefulness for preventing diseases and decreasing the need for antibiotics. For example, contagious bovine pleuropneumonia, caused by the bacterium *Mycoplasma mycoides*, remains an economically important disease of cattle in sub-Saharan Africa that often necessitates considerable antibiotic use [44]. The currently available live vaccine has limited efficacy and duration of immunity, and potentially severe side effects [44]. The development of a safer and more efficacious vaccine is complicated by a variety of factors such as a limited understanding of host–pathogen interactions including basic pathophysiological and immunological processes during infection, a suboptimal challenge model that complicates data interpretation, and the possibility of considerable additional regulatory requirements for the licensing of genetically modified live vaccines [44].

Although not likely to directly reduce antibiotic consumption, the European Commission’s project to generate an improved classical swine fever vaccine (CSFV-GODIVA project) also provides useful insights into the types of challenges associated with many current veterinary vaccines. Specifically, the project developed a new modified live classical swine fever marker vaccine that overcame many limitations of the previously existing vaccines with regard to the ability to distinguish vaccinated from naturally infected animals, the immunogenicity of the vaccine, and the suitability for oral applications, in particular for mass-scale wildlife vaccination [45]. The development of a safe and effective vaccine against African swine fever has been similarly complicated by various factors such as a limited understanding of the immune response to infection, strain-dependent effects of gene deletions on virulence attenuation and protection, a dearth of small-animal and in vitro models, and a complex disease epidemiology. Modified live vaccines against this viral disease have various drawbacks, including severe side-effects and the potential for undetected, subclinical infections in vaccinated animals that may result in viral shedding and can also lead to recombination between field and vaccine strains [46]. The development of African swine fever subunit vaccines, on the other hand, has been hampered by suboptimal delivery or vector systems that often fail to induce a protective immunity [46].

As can be inferred from these examples, a variety of challenges are shared broadly across different veterinary vaccines. Additional file 1 synthesizes some of these general limitations associated with many current veterinary vaccines, based on an assessment of an OIE ad-hoc Group on Prioritisation of Diseases for which Vaccines could Reduce Antimicrobial Use in Animals (see next section) and a review of research gap data for more than 50 infectious diseases of animals produced by expert groups and captured in DISCONTOOLS, a database created as part of the Action Plan of the European Technology Platform for Global Animal Health and funded under the EU 7th framework program [47].

As shown in Additional file 1, current veterinary vaccines often fall short with regard to efficacy, safety and/or user-friendliness. The reasons why veterinary vaccines may have limited efficacy are quite varied. In some cases [e.g., *Streptococcus suis*, swine influenza virus, *Haemophilus parasuis*, *Eimeria* species (Additional file 2)], the vaccine strain may not be a good match for the field strain. For instance, the pathogen may be evolving quickly and the vaccine may not be updated to confer protection against current strains [e.g., infectious bronchitis virus, porcine reproductive and respiratory syndrome virus (PRRSV) (Additional file 2)], or it may only protect against a limited subset of strains [e.g., PRRSV, *Actinobacillus pleuropneumoniae* (Additional file 2)]. In other cases, protection after vaccination may be short-lived and require frequent booster vaccinations [e.g., *Clostridium perfringens,* bovine respiratory syncytial virus (Additional file 2 and DISCONTOOLS)]. In some cases vaccines do not generate a protective immune response at all (e.g., African swine fever virus, see DISCONTOOLS). This is most commonly the case for inactivated or subunit vaccines. Because these vaccines are not actively replicating in the host cells they tend to only induce humoral immune responses, even though cellular immune responses are vitally important for effective protection against many pathogens. Vaccine efficacy depends on the existence of an intact and properly functioning immune system, and administration has to be timed correctly to account for the lag period required to develop a protective immune response. Eliciting protective immune responses in young animals tends to be particularly challenging because the immune system is still developing, and because maternal antibodies can interfere with the development of protective immunity. Vaccination against diseases that require protective immunity in young animals can therefore be particularly challenging [e.g., infectious bursal disease virus (Additional file 2)]. In addition, many veterinary vaccines effectively reduce the severity and economic impact of the disease, but do not fully prevent infection and shedding and therefore do little to reduce disease incidence [e.g., *M. hyopneumoniae* (Additional file 2)]. In some cases, vaccination can actually increase the survival time for infected animals and therefore enhance opportunities for disease transmission. Vaccines are also not available for all economically important veterinary diseases, including many parasitic infections as well as secondary bacterial infections, diseases of “minor species” such as bees, and diseases that have been largely eliminated by management practices but that are recently increasing in incidence [e.g., liver flukes, nematodes, varroa mites, omphalitis, airsacculitis, cellulitis (Additional file 2 and DISCONTOOLS)].

A variety of safety issues are shared by various current veterinary vaccines. Potentially severe side-effects are a concern for many veterinary vaccines, in particular for attenuated-live vaccines and certain adjuvants, and can result in abortions, malformations and deaths (e.g., contagious bovine pleuropneumoniae, African horse sickness, lumpy skin disease, rift valley fever virus, see DISCONTOOLS). Even for vaccines with less dramatic side-effects, such as coccidia vaccines, productivity losses can be impactful and discourage routine use. Attenuated live vaccines can also carry a risk of reversion to virulent wild type strains, particularly when the molecular changes responsible for the attenuation of the vaccine strain have not been well-characterized (e.g., bovine respiratory syncytial virus, African horse sickness virus, bluetongue virus, PRRS, see DISCONTOOLS). Similarly, some live vaccines carry a risk of horizontal and/or vertical transmission and outbreaks caused by vaccine strains have been described (e.g., orf, PRRS, rift valley fever, see DISCONTOOLS). Finally, for some diseases prior vaccination can actually lead to an exacerbation of clinical symptoms after infection (e.g., bovine respiratory syncytial virus, *Mycoplasma bovis*, see DISCONTOOLS). The immunological reasons for this exacerbation are generally not well understood, but are thought to be due to a shift in immune response after vaccination (e.g., towards Th2-type responses).

User-friendliness issues can further limit the usefulness of current vaccines. For instance, mass vaccination through spray, drinking water or bait can significantly reduce labor costs, directly deliver vaccines to mucosal surfaces, and may be the only feasible strategy in certain situations such as widespread vaccination of wildlife reservoirs. Unfortunately, immunological processes such as the development of tolerance after mucosal antigen exposure (discussed in detail in section below) complicate the development of vaccines for mass application and most current inactivated, subunit and DNA vaccines require administration by injection. The potential for user errors can also limit vaccine usefulness, for instance errors in vaccination route, dose and frequency of vaccination, and in proper vaccine handling. Some vaccines, in particular certain attenuated live vaccines, are of limited stability, leading to cumbersome cold storage requirements and short shelf life, which can complicate vaccine use under field conditions (e.g., foot and mouth disease virus, *Theileria*, see DISCONTOOLS). Vaccine manufacturing quality can also be a challenge, in particular with certain autogenous or regional vaccines. In some cases, limited diagnostic capabilities can make it difficult to verify vaccinated animals have mounted a protective immune response, which can hinder both the effective use of existing vaccines and the development of new ones (e.g., mastitis vaccines, bovine respiratory syncytial virus, paratuberculosis). Marker vaccines allow vaccinated animals to be distinguished from naturally infected animals, a vital distinction for many disease control and eradication programs. Unfortunately, marker vaccines are currently only available for a subset of animal diseases and the development of additional vaccines will likely be complicated by the need for sensitive and specific diagnostic tests that can be used in combination with the marker vaccine. Commercial interest in developing vaccines for animal diseases is a critically important driver of innovation, but in reality often remains limited. Reasons include the relatively high cost of production for many vaccines, the costs and time associated with laborious administration protocols, in particular if multiple booster vaccinations are required, and the limited cost-effectiveness compared to other available control options including antibiotics. Regulatory restrictions, for instance related to novel vaccine technologies such as genetically modified live vaccines, can further limit commercial interest in vaccine development.

## Investment decision-making in the research and development of veterinary vaccines

The development of veterinary vaccines requires considerable time and resource investments, which pharmaceutical companies could dedicate to other products that may be deemed to generate a higher return on investment. Factors considered by the pharmaceutical industry in the decision to develop a vaccine go beyond demonstration of efficacy. They include unmet needs of the animal agriculture industry, market potential, the probability of success and the time to market as well as the emergence of antibiotic resistance. Because of the substantial time required for research, development and regulatory approval, these decisions rely on a prediction of the situation at the time of and subsequent to expected market entry. Uncertainty in these predictions can have a stifling effect on pharmaceutical research and development investments. Importantly, the current and future availability of other safe and effective management options for the disease, including the availability of antibiotics, affects this prediction and therefore also has to be considered. In fact, the economic attractiveness of vaccines is partially dependent on the cost of alternative disease management options, including the cost of antibiotics where available, although direct and indirect benefits on human health including potential food safety improvements may also be factored into the consideration.

The development strategy for new vaccines should therefore be aimed at meeting the needs of the animal production industry and consider issues such as the length of and common animal health challenges encountered during animal production cycles, although public health benefits also should be considered. Combination vaccines that target multiple pathogens are one commonly used strategy to overcome the narrow spectrum of most vaccines, which is generally much narrower than that of antibiotics. Polyvalent and combination vaccines therefore may be more attractive alternatives and more effective in reducing the need for antibiotics than monovalent vaccines. The development of new safe and effective adjuvants or the combination of vaccines with immune modulators may be a promising strategy for overcoming limitations in vaccine efficacy, in particular for relatively short-lived species such as poultry. Practical considerations, for instance the feasibility of vaccine administration to individual animals, also have important strategic implications and oral vaccines that lend themselves to mass vaccination tend to be particularly appealing to industry—if they can be developed successfully. Species-specific factors, such as the innate ability to react to immunological triggers [e.g., lipopolysaccharide (LPS)] have to be considered as well. In fact, because of the vast physiological and immunological differences among animal species and existing gaps in basic knowledge, adapting vaccines to new species may be challenging and resource-intensive. Vaccines for minor species may pose a particular challenge in that regard- and “minor” species such as sheep and goats may in actuality constitute very large and important parts of the animal populations in some countries. Public–private partnerships may be a strategy to incentivize the development of vaccines that would otherwise not be a high priority for the pharmaceutical industry because they can reduce research and development costs, limit the associated risks, and allow public and private partners to leverage their unique strengths. In fact, European Commission funding for the CSFV-GODIVA project demonstrates how public funding can drive the development of safer and more effective vaccines, even in situations such as classical swine fever where vaccine use is severely restricted by government regulations in the traditional major animal health product markets.

Close collaboration between private industry, government, and academia is important to ensure that research efforts are complimentary, and that each party’s unique strengths will foster progress towards the common goal of developing vaccines effective in reducing the need for antibiotics; for instance, academic (and in some cases government) partners may be best equipped to conduct basic research (e.g., on species-specific differences in immune responses) and to develop “companion technologies” such as diagnostic tests or adjuvants in an efficient and cost-effective manner. These technologies may prove critical to the commercialization of a new vaccine, but reliable technology transfer strategies and close alignment with the industry will be important to assure their proper functioning in conjunction with the newly developed vaccine. On the other hand, funding agencies may be reluctant to fund the types of large-scale animal trials needed to demonstrate vaccine efficacy, and academic researchers may have to depend on the pharmaceutical industry to conduct these types of studies. Close alignment between academic and industry researchers can help here as well—for instance by ensuring initial studies by academic institutions are appropriately informing subsequent larger animal trials, and are ideally designed and conducted in ways that allow the data to be used as part of regulatory submissions.

Regulatory approval processes also have an important impact on the decision whether to invest in the research and development of a new vaccine. For instance, pharmaceutical companies typically seek to license a given product in all of the major animal health markets. Harmonization and streamlining of regulatory approval pathways across countries and regions can reduce the associated development cost and make the product more attractive to investors within and outside of the pharmaceutical industry. Regulatory strategies such as early consultations with regulatory officials can further reduce the overall research and development cost and allow for the rapid development of a vaccine to address new animal disease challenges. In some situations, additional, more flexible regulatory pathways may need to be considered to address specific challenges, such as disease challenges that are specific to a limited geographic region or that require vaccination of certain wildlife species.

Lastly, the development of new veterinary vaccines has to be considered within the broader context of animal health and microbial ecologies. For instance, vaccination against one pathogen may have unintended implications for the incidence of other pathogens through processes such as niche alteration. Similarly, as one disease is increasingly controlled by vaccination others may become more important to the animal production industries and may begin to drive antibiotic consumption. At the same time, it will be impossible to develop efficient vaccines for all animal diseases for which antibiotics are used. Therefore, limited research and development resources have to be targeted to priority diseases to ensure maximum impact.

## Prioritization of diseases for which vaccines can reduce antibiotic use

Several OIE Member Countries and organizations have requested guidance on the prioritization of investments that may reduce the need for antimicrobial use in animals, in particular in intensive poultry, pig and fish production systems that are projected to expand globally. In April 2015, the OIE convened an ad hoc Group of relevant experts to provide direction to policy makers regarding investments in vaccine research, prioritizing diseases and syndromes with highest impact on antimicrobial consumption [48]. In order to identify infections where new or improved vaccines would have the maximum potential to reduce antibiotic use, a number of key questions were considered:

1. What are the most prevalent and important bacterial infections in chickens and swine; in which commonly farmed fish species is antibiotic use common, and which bacterial infections are prevalent in those fish species?
2. Which common non-bacterial infections, for instance caused by protozoal or viral pathogens, trigger empirical antibiotic treatments in chicken, swine and fish and also frequently result in bacterial co-infections?
3. For each of the identified diseases and syndromes, is associated antibiotic use high, medium or low relative to total antibiotic use in that animal species?
4. For each of the identified diseases and syndromes, are vaccines available and what is their effectiveness?
5. What is the potential for new or improved vaccines to reduce the need for antibiotic treatment?

With the exception of vaccine design, factors that influence the utilisation of a vaccine were considered out of scope for the task of this group. Also considered out of scope were autogenous vaccines, primarily because of their lack of broad applicability across time and space, registration variability, and the absence of key efficacy data.

The fundamental difference in spectrum between antibiotics and vaccines presented a key challenge to the identification of promising candidates to reduce antibiotic consumption. First line antibiotic use in animal production is often empirical, based on clinical symptoms, such as diarrhoea or respiratory signs, and guided by experience. In contrast, as discussed above, current vaccines tend to have a narrow spectrum that is limited to specific pathogens or pathogen strains. Significant data gaps further complicated the prioritization. For example, at the time of the meeting, a current list of all available vaccines globally with marketing authorisation was not available. Comprehensive data on antibiotic consumption for the various infections in the animal species, and the relative incidence of these infections worldwide were also sparse. The prioritization therefore relied on expert opinion to close key data gaps.

The group agreed that effective vaccines for the diseases listed in Additional file 2 could significantly reduce the need to use antibiotics in swine, poultry, and fish farming. However, significant scientific and technical hurdles exist, and an overarching investment in vaccine research could have a significant positive effect, particularly if it addressed the following four priority areas:

1. Maternal antibody interference.
2. Cross-protection or inclusion of relevant strains in vaccine formulations.
3. Occurrence of immunological interference in multivalent vaccines.
4. Innovative delivery systems to enable mass vaccination.

The report was distributed for consideration to research funders and global animal health research organizations (e.g. STAR-IDAZ).^2^ The group also recommended that global vaccine research networks be created to provide resources and expertise in the development of vaccines for these critical diseases [49].

Specific examples of recent scientific progress are provided in part 2 of this manuscript.

## Conclusions

Vaccines are proven strategies for the prevention or control of infectious diseases in animal populations. Therefore, they are promising alternatives that can reduce the need to use antibiotics in food-producing animals and their direct mitigating impact on antibiotic consumption has been demonstrated in a number of studies, even though the relationship between antibiotic use and vaccination is not in all cases clear-cut. The ideal vaccine is safe, effective against a broad range of pathogens, and easily adapted to mass-application. At the same time, it is cheap to produce and use, easy to register across key jurisdictions, and generates durable protection, ideally after a single administration.

Existing vaccines still fall short of these ideals. In fact, many current vaccines have a number of shortcomings with regard to safety, efficacy and/or user-friendliness that limit their ability to replace antibiotic use. Overcoming these challenges will take close collaboration and innovative new approaches. Public–private partnerships represent one promising governing structure for assuring such close collaboration across public and private sectors. Investments in basic and applied research are equally needed to overcome these challenges, and research needs will have to be prioritized to ensure scarce resources will be preferentially dedicated to areas of greatest potential impact. Research to characterize and quantify the impact of vaccination on antibiotic use is equally needed.

Yet, some data demonstrating the ability of vaccines to reduce antibiotic consumption are already available. Similarly, as highlighted in part two of this two-part manuscript, key research breakthroughs and a number of highly promising vaccination approaches are already in development. These include new oral vaccines based on bacterial spores, live vectors, or new delivery strategies for inactivated oral vaccines; they also include new vaccination strategies in-ovo, combination vaccines that protect against multiple pathogens, the use of recent biotechnological advances, and comprehensive approaches to manage diseases caused by ubiquitous pathogens.

Therefore, further reductions in the need for antibiotic use through the use of new vaccines are all-but-certain, and investments in research and development of new vaccines will be vital for the sustained success of animal agricultural production around the world.

## Additional files

**Additional file 1.** **Limitations associated with current veterinary vaccines and approaches for overcoming these challenges.** Analysis based on DISCONTOOLS.

**Additional file 2.** **Infections by species where new/improved vaccines would significantly reduce the need for antibiotic use.** Results of OIE ad-hoc group on prioritization of diseases for which vaccines could reduce antibiotic use.

## Footnotes

1 Alternatives to antibiotics, in this context, are new options and alternative strategies for the prevention and treatment of animal diseases that reduce the use of medically important antibiotics, including those deemed critically important for human health, in animal agricultural production.

2 Global Strategic Alliances for the Coordination of Research on the Major Infectious Diseases of Animals and Zoonoses.
