# Supplementary material for: Vaccines as alternatives to antibiotics for food producing animals. Part 1: challenges and needs
Source: Vet Res. 2018 Jul 31;49:64. doi: 10.1186/s13567-018-0560-8 (PMC6066911; doi:10.1186/s13567-018-0560-8)
Supplement: Supplementary file 1 — Additional file 1. Limitations associated with current veterinary vaccines and approaches for overcoming these challenges. Analysis based on DISCONTOOLS. [file 13567_2018_560_MOESM1_ESM.docx]

**Additional file 1 Limitations associated with current veterinary vaccines and approaches for overcoming these challenges [47, 48] ^[[1]](#endnote-1)^**

| **Limitations associated with current vaccines** | **Examples** | **Reasons / implications** | **Approaches for overcoming challenges** |
| --- | --- | --- | --- |
| 1. **Efficacy** |  |  |  |
| Vaccine protects only against limited set of field strains | bluetongue; coccidiosis; leptospirosis; mycoplasma bovis; PRRS; PCV 2; swine influenza virus; *E. coli*; infectious bronchitis virus; *A. pleuropneumoniae* | - Surface antigen diversity - Limited cross-protection - High mutation rate, genetic drift - Reassortments - Immune evasion - Emerging strains - Vaccines not available for all field strains | - Polyvalent vaccines - Serial vaccinations - Recombinant protein or subunit vaccines - Updated vaccines - Surveillance of field strains; diagnostics - Annual vaccination |
| Short-lived immune protection; frequent boosters needed | many inactivated, subunit or recombinant vaccines; orf vaccines; *clostridium perfringens* vaccines against necrotic enteritis | - cellular & mucosal immunity pivotal for protection, but vaccines elicit primarily humoral response - Variable response to vaccination | - Vectored, DNA or modified live vaccines - Adjuvanted live attenuated vaccines - Research on immune response to infection - Development of new delivery systems that stimulate cell-mediated immunity - combination with other vaccines to improve feasibility |
| Ineffective immune protection | many inactivated, subunit or recombinant vaccines; vaccines for multifactorial infections | - Lack of cellular / mucosal immune responses after vaccination - low level of opsonizing antibodies at site of infection (e.g., udder lumen) - recurrent infections despite neutralizing antibodies because of mismatch between vaccine and field strain - complex disease syndrome caused by interaction of multiple pathogens, only some of which are included in vaccine | - Potent adjuvants - Vectored, DNA or modified live vaccines - Research on disease pathogenesis and/or virulence factors - Administration routes that ensure uniform exposure - More effective antigens - Improved understanding of adaptive and non-specific immune responses |
| Limited protection in young animals | paratuberculosis; bovine respiratory syncytial virus; coccidiosis;; infectious bursal disease; haemophilus parasuis; swine influenza virus; *E. coli* | - Interference with maternal antibodies - Vaccination does not fully prevent shedding, allowing transmission to naïve animals - Narrow window of opportunity to establish protective immunity | - Vaccination protocols that avoid interference of maternal antibodies paired with appropriate management practices - Maternal vaccines that provide lactogenic immunity in neonatal animals |
| Vaccine reduces losses but not disease incidence | bovine tuberculosis; paratuberculosis; coccidiosis; BVD; chlamydiosis; environmental mastitis, salmonellosis; *staphylococcus* aureus; A. pleuropneumoniae; *Mycoplasma* in swine | - Vaccine does not fully prevent infection & shedding - Vaccinated animals may not be distinguishable from naturally infected animals - transmission may continue to occur - persistently infected animals may continue to be generated - vaccine may increase survival of shedders - potential latency & reactivation in some cases | - integration of vaccination in disease control/eradication strategy - DIVA/marker vaccines - more effective vaccines - workable definition of vaccine effectiveness |
| No commercial vaccine available | many parasitic infections; secondary bacterial infections; diseases in minor species (e.g., bees); diseases that were largely eliminated by management practices (e.g., *Brachyspira*) | - pathogenesis and/or immune response to infection incompletely understood - inability to elicit protective immune response - lack of financial incentives | - basic research on pathogenesis and immune response - identification of protective antigens - research on cost-effectiveness |
| 1. **Safety** |  |  |  |
| Side-effects of vaccination  (e.g., abortion, malformations, deaths, or productivity losses) | brucellosis; coccidiosis; lumpy skin disease; chlamydiosis; rift valley fever | - Residual virulence - Pathogenic potential - Side-effects of vaccine strain infection / circulation of oocytes - Side-effects of adjuvants - For some (zoonotic) vaccines, safety concerns for humans administering vaccine | - Recombinant and vectored vaccines; inactivated vaccines - Safer adjuvants - Research into pathogenicity traits |
| Risk of reversion to wild type | Several attenuated live vaccines, for instance bovine respiratory syncytial virus; PRRS; bluetongue | - Genetic event (e.g., point mutations, homologous recombination, reassortment) effectively reverses attenuating mutations - Attenuated live virus regains virulence characteristics | - Non-replicating vaccines (e.g., DNA, vectored, subunit or inactivated vaccine) - Research on pathogenicity and virulence traits - Better characterization of vaccines |
| Risk of vaccine infections or outbreaks | Various live vaccines, for instance parapox; PRRS; Rift valley fever; coccidiosis; bluetongue; paratuberculosis | - Vaccine-mediated outbreaks - Horizontal and/or vertical transmission - Fetal abnormalities / abortions / stillbirths - Vector-born spread - Increased longevity of shedders may increase disease spread in naïve herd or flock | - Non-replicating vaccines - Vaccines with less/no shedding, lower viremia or bacteremia and lower transmission risk - Management of transmission risk, timing of infection, etc. |
| Disease exacerbation in vaccinated animals | bovine respiratory syncytial virus; mycoplasma bovis; swine influenza virus | - Primed immune response has detrimental impact on disease progression; for instance Th2 type immune response | - basic research to understand immune response to infection and mechanism of disease exacerbation |
| 1. **Ease of use** |  |  |  |
| Limited opportunity for mass vaccine application | Most inactivated, subunit and DNA vaccines; *Clostridium perfringens* vaccines against necrotic enteritis | - administration through injection - limits ability to vaccinate wildlife reservoirs - leads to considerable labor costs | - Live attenuated or vectored vaccines - New, more effective application methods (ideally by spray, drinking water or bait) - Oral vaccines |
| Vaccine stability / cold storage requirement / special management requirements | Foot and mouth disease; theileria; Lawsonia | - Heat labile pathogen basis for attenuated live vaccine - Interference of antibiotics with vaccination efficiency | - More stable vaccine – e.g., empty capsids, subunit vaccines |
| Issues with vaccine quality | many autogenous vaccines; some regional vaccines | - Manufacturing challenges | - Improved manufacturing |
| Limited ability to verify vaccine efficacy | bovine respiratory syncytial virus; mastitis vaccines; orthopox and parapox viruses; paratuberculosis; | - Protection mediated through cellular and mucosal immunity while presence of neutralizing antibodies not protective, resulting in inability to detect protective immune response - Unclear impact of unspecific immune responses - Questionable vaccine efficiency requirement for herd immunity - Limited ability to screen vaccine candidates | - New diagnostic assays - Field studies - Definition of vaccine efficacy - Research on mechanism of protective immune responses |
| Distinction between vaccinated and infected animals | bluetongue; bovine tuberculosis; brucellosis; BVD; contagious bovine pleuropneumoniae; lumpy skin disease; Mycoplasma bovis; PCV 2; peste des petits ruminants | - Infections with vaccine strains indistinguishable from field strains by diagnostic tests | - DIVA/ marker vaccines and assays - Research on virulence factors and protective antigens |
| Regulatory restrictions | Various foreign animal diseases (e.g., classical swine fever; foot and mouth disease); pathogens with multiple disease manifestations | - Restrictions on use in disease free countries or countries with active eradication program - Vaccine with no label claim for disease but potential efficacy ( e.g, pneumonic *cases* of *Pasteurella multocida*, with vaccine claim for atrophic rhinitis) - Restrictions related to vaccine technology (e.g., GMO vaccines) | - DIVA/marker vaccines - Economic incentives for vaccine development / stockpiling - Regulatory approaches |
| Limited commercial interest | many vaccines, particularly subunit and inactivated vaccines | - high cost of production - numerous boosters - laborious administration - limited cost-effectiveness - more expensive than alternative management strategies - limited market size - use restrictions - no animal health impact (food safety / public health motivated) | - cheap, single dose vaccines - polyvalent vaccines - combination vaccines - cost-effectiveness research - public-private partnerships - incentives for vaccine research and development - new production and administration methods |

1. Analysis is based on data from DISCONTOOLS, a database to identify research gaps on vaccines (as well as pharmaceuticals and diagnostics) for the control of 52 priority animal diseases, and results of the OIE ad hoc group for the prioritization of diseases for which vaccines could reduce antimicrobial use in animals (see Additional file 2). [↑](#endnote-ref-1)
