# Supplementary material for: Vaccines as alternatives to antibiotics for food producing animals. Part 1: challenges and needs
Source: Vet Res. 2018 Jul 31;49:64. doi: 10.1186/s13567-018-0560-8 (PMC6066911; doi:10.1186/s13567-018-0560-8)
Supplement: Supplementary file 2 — Additional file 2. Infections by species where new/improved vaccines would significantly reduce the need for antibiotic use. Results of OIE ad-hoc group on prioritization of diseases for which vaccines could reduce antibiotic use. [file 13567_2018_560_MOESM2_ESM.docx]

**Additional file 2 Infections by species where new/improved vaccines would significantly reduce the need for antibiotic use [48].**

| **Key syndrome** | **Primary pathogen(s) (disease)** | | | **Antibiotic use** | **Commercial* vaccine exists** | **Major constraints to use of vaccine / vaccine development** | | | **Vaccine research priority** |
| --- | --- | --- | --- | --- | --- | --- | --- | --- | --- |
| **Chickens** | | | | | | | | | |
| Systemic (Broilers) | *Escherichia coli  (Yolk sac infection, airsacculitis, cellulitis)* | | | High | Yes | - Omphalitis: secondary bacterial infection – not a disease one can immunize against - Strain coverage limited - Airsacculitis, cellulitis: vaccines available, e.g. live aerosol vaccine. However, Serotype coverage limited and field efficacy variable | | | High |
|  | *Infectious Bursal Disease virus (secondary bacterial infections)* | | | Medium | Yes | - Issues with vaccine application - Short window of opportunity to vaccinate - Maternal antibody interference | | | Medium |
| Systemic (Breeders, Layers) | *Escherichia coli (airsacculitis, cellulitis, salpingitis and peritonitis)* | | | High | Yes | - Strain coverage limited | | | High |
| Enteric  (Broilers, Breeders, and Layers) | *Clostridium perfringens,* type A (necrotic enteritis) | | | High | Yes | - Toxoid vaccine for layers providing only short-lasting passive immunity - Research needed to achieve active immunity. - Improved and/or more convenient (mass vaccination) vaccine needed for broilers | | | High |
|  | *Eimeria* species  Coccidiosis (secondary bacterial infections) | | | High | Yes | - Lack of cross-protection between species - Strains must be matched to infectious agent for some species - Many current vaccines are not attenuated and can produce low dose infection - Sub-unit vaccines have not been successful | | | High |
|  | *Infectious Bronchitis virus (secondary bacterial infections)* | | | Medium | Yes | - Issues with strain matching and strain coverage - High mutation rate of virus | | | Medium |
| **Swine** | | | | | | | | | |
| Systemic (respiratory) | *Streptococcus suis* | | | *High* | Yes | - Strain coverage too narrow - Lack of cross-protection - Poor immunogenicity due to being a capsule based vaccine | | | High |
|  | *Haemophilus parasuis* | | | *Medium* | Yes | - Serotype specific with variable cross-protection - Maternal antibody interference | | | Medium |
| Respiratory | *Pasteurella multocida (for pneumonic disease)* | | | *High* | No | - No vaccine with approved label claim for pneumonia (There is a vaccine for atrophic rhinitis) | | | High |
|  | *Mycoplasma hyopneumoniae* | | | *High* | Yes | - Does not completely prevent lung lesions - Animals continue to shed pathogen - Diagnostics not always accurately done | | | Low |
|  | *Actinobacillus pleuropneumoniae* | | | *High* | Yes | - Limited coverage - Good immunity only if serotype specific - Sub-unit vaccine which affords cross-protection | | | High |
|  | *Porcine Reproductive and Respiratory Syndrome virus (secondary bacterial infections)* | | | *High* | Yes | - Strain coverage limited - High virus mutation rate - Modest cross-protection - Vaccine evasion | | | High |
|  | *Swine Influenza Virus (secondary bacterial infections)* | | | *High* | Yes | - Strain matching - Vaccine-associated enhanced respiratory disease (VAERD) - Lack of cross-protection - Efficacy in piglets limited | | | High |
| Enteric – neonatal | *Escherichia coli* | | | *High for the syndrome, Low for E. coli* | Yes | - Maternal vaccine provides effective lactogenic immunity - Coverage of enterotoxigenic E. coli may occasionally need to be updated | | | Low |
| Enteric (weaners/finishers) | *Escherichia coli* | | | *High* | Yes | - Maternal antibody interference - Short window for induction of immunity | | | High |
|  | *Lawsonia intracellularis* | | | *High* | Yes | - Other pathogens in the syndrome (Brachyspira) not included - Antibiotic-free window for vaccination required (live attenuated oral vaccine) | | | Low  (see also Brachyspira) |
|  | *Brachyspira spp  B. hyodysenteriae,  B. pilosicoli* | | | *Medium-high* | No | - Low current research investment as changes in husbandry largely eliminated the disease - Technical barriers to vaccine development | | | High |
|  | *Rotaviruses (secondary bacterial infections)* | | | *High* | Yes | - Reasons limiting wider adoption unknown | | | High |
| **Freshwater cyprinids** | | | | | | | | | |
| Systemic bacterioses | *Aeromonas hydrophila* and other species | | High | | No | | - Disease is caused by a wide range of serotypes | High | |
| Dermal bacterioses / red spot disease | *Pseudomonas* spp. | | High | | No | | - Disease is caused by a range of species and wide range of strains and serotypes | High | |
| Columnaris | *Flavobacterium columnare* | | Medium | | Yes | | - Limited uptake by some countries for unknown reasons | Low | |
| **Freshwater cichlids** | | | | | | | | | |
| Systemic/dermal bacterioses | *Aeromonas hydrophila* and other species | Medium | | | No | | - Disease is caused by a range of species and wide range of strains and serotypes | Medium (not low because of projected increase in production) | |
|  | *Streptococcus inae,  S. agalactiae* | Medium | | | Yes | | - Industry awareness of need is low (first vaccine only became recently available) | Medium | |
| **Freshwater salmonids** | | | | | | | | | |
| Systemic bacterioses | *Aeromonas salmonicida, Yersinia rukerii, Flavobacterium psychrophilum,  Vibrio anguillarum* | Medium | | | Yes  (multivalent, injectable) | | - cost of vaccine is high relative to harvest value | Low | |
| **Marine salmonids** | | | | | | | | | |
| Salmon Rickettsia Syndrome | *Piscirickettsia salmonis* | Medium | | | Yes | | - Multivalent vaccine which provides low protection for *P. salmonis* compared to other pathogens included in the vaccine. | Unknown because the recent introduction of an oral monovalent vaccine booster may improve the level of protection | |
| **Other marine fish** | | | | | | | | | |
| Systemic / dermal bacterioses | *Vibrio* spp., *Photobacterium* spp. | Medium | | | Yes | | - Disease is caused by a wide range of serotypes - Industry awareness is low in some countries | High | |
|  | *Streptococcus* spp. | Medium | | | Yes | | - Disease is caused by a wide range of serotypes - Industry awareness is low in some countries | High | |
| **Catfish** | | | | | | | | | |
| Systemic | *Edwardsiella ictaluri,  E. tarda* | Medium | | | Yes  (for Channel catfish) | | - Vaccines are not available for African catfish (an important farmed species) - Vaccines have very recently become available for Tra catfish and yet to be adopted by the industry | High  (for African catfish) | |
| Systemic | *Aeromonas hydrophila* and other species | Medium | | | No | | - Disease is caused by a wide range of serotypes | High | |
